# Tree Species Traits Influence Soil Physical, Chemical, and Biological Properties in High Elevation Forests

**DOI:** 10.1371/journal.pone.0005964

**Published:** 2009-06-18

**Authors:** Edward Ayres, Heidi Steltzer, Sarah Berg, Matthew D. Wallenstein, Breana L. Simmons, Diana H. Wall

**Affiliations:** 1 Natural Resource Ecology Laboratory, Colorado State University, Fort Collins, Colorado, United States of America; 2 Department of Biology, Colorado State University, Fort Collins, Colorado, United States of America; University of Oxford, United Kingdom

## Abstract

**Background:**

Previous studies have shown that plants often have species-specific effects on soil properties. In high elevation forests in the Southern Rocky Mountains, North America, areas that are dominated by a single tree species are often adjacent to areas dominated by another tree species. Here, we assessed soil properties beneath adjacent stands of trembling aspen, lodgepole pine, and Engelmann spruce, which are dominant tree species in this region and are distributed widely in North America. We hypothesized that soil properties would differ among stands dominated by different tree species and expected that aspen stands would have higher soil temperatures due to their open structure, which, combined with higher quality litter, would result in increased soil respiration rates, nitrogen availability, and microbial biomass, and differences in soil faunal community composition.

**Methodology/Principal Findings:**

We assessed soil physical, chemical, and biological properties at four sites where stands of aspen, pine, and spruce occurred in close proximity to one-another in the San Juan Mountains, Colorado. Leaf litter quality differed among the tree species, with the highest nitrogen (N) concentration and lowest lignin∶N in aspen litter. Nitrogen concentration was similar in pine and spruce litter, but lignin∶N was highest in pine litter. Soil temperature and moisture were highest in aspen stands, which, in combination with higher litter quality, probably contributed to faster soil respiration rates from stands of aspen. Soil carbon and N content, ammonium concentration, and microbial biomass did not differ among tree species, but nitrate concentration was highest in aspen soil and lowest in spruce soil. In addition, soil fungal, bacterial, and nematode community composition and rotifer, collembolan, and mesostigmatid mite abundance differed among the tree species, while the total abundance of nematodes, tardigrades, oribatid mites, and prostigmatid mites did not.

**Conclusions/Significance:**

Although some soil characteristics were unaffected by tree species identity, our results clearly demonstrate that these dominant tree species are associated with soils that differ in several physical, chemical, and biotic properties. Ongoing environmental changes in this region, e.g. changes in fire regime, frequency of insect outbreaks, changes in precipitation patterns and snowpack, and land-use change, may alter the relative abundance of these tree species over coming decades, which in turn will likely alter the soils.

## Introduction

Organic matter inputs to soil come primarily from plants, for example via rhizodeposition and litter fall. In addition, plants take up a range of soil resources, such as water, nitrogen (N), and phosphorus, and as a result plants strongly influence physical, chemical and biological properties of soil. However, since plants exhibit broad variation in their natural history and physiology [1], [2], it is likely that differences in plant species traits will create distinctive soil environments and biotic communities [3]. For instance, plant species differ in the quality and quantity of their inputs to soil, root architecture, and nutrient requirements [4], [5], [6], [7], [8], [9].

Previous studies have demonstrated that different plant species influence the soil environment in different ways, which relates to plant species traits. Pot and field experiments have shown that soil temperature, soil moisture, and microbial physiology and community composition differ in soil planted with different grassland plant species [2], [10], [11], [12]. Indeed, studies of grassland plant biodiversity on soil properties frequently observed stronger effects of plant community composition than species richness, indicative of the importance of plant species traits for soil and ecosystem properties [13], [14]. Similarly, soil moisture, pH, microbial biomass, respiration rate, and N availability differed among tree species grown in pots, and the difference could in part be attributed to differences in tree species growth rates [15], [16], [17]. Large-scale common garden experiments have also observed differences in soil properties, such as pH, carbon (C) content, inorganic N concentrations, and earthworm biomass, among tree species, which related to plant species traits including leaf litter calcium concentration and natural history [18], [19], [20], [21]. These plant species effects on soils can have positive, neutral, or negative feedbacks to the plant species, which depends in part on whether the plant is an early- or late-successional species [22], [23], [24], [25].

Here we assessed whether three tree species in the San Juan Mountain Range of the southern Rocky Mountains had species-specific impacts on soil physical, chemical, and biological properties. Trembling aspen (*Populus tremuloides*), lodgepole pine (*Pinus contorta*), and Engelmann spruce (*Picea engelmannii*) are common tree species in this region and throughout much of western USA and Canada, and vary in traits such as litter chemistry and natural history [26]. In addition, a previous study of stands of these tree species indicated that they influence soil N cycling in different ways, which related to leaf litter quality, although these differences may have been influenced by factors other than tree species, e.g. elevation, stand age, and climate, which also varied among stands [26]. Moreover, the impact of these tree species on soil biota and on other soil properties remains unknown.

The relative abundance of aspen, pine, and spruce may change over coming decades as they face a variety of local, regional, and global environmental changes, including rising temperatures, insect outbreaks, multi-year droughts, decreased snow pack, aspen die-offs (cause currently unknown), land-use change, and altered fire frequency [27], [28], [29], [30], [31], [32], [33]. Therefore, an understanding of the different effects these tree species have on ecosystem properties and processes may assist with forecasting changes in ecosystem properties and processes in this region. We hypothesized that differences in species traits among aspen, pine and spruce would influence soil physical, chemical, and biological properties. Specifically, we expected aspen stands to have higher temperatures due to their open structure, which, combined with higher quality litter, would result in increased soil respiration rates, N availability, and microbial biomass, and differences in soil faunal community composition.

## Materials and Methods

The study was located in the San Juan Mountains of southwest Colorado, USA, on the western slope of the southern Rocky Mountains. Our study focuses on the forests south of the town of Silverton and elevation in this region ranges from about 2450 m in the valleys up to 4300 m. In Silverton, elevation 2825 m, which is about 11 km from the field sites, mean annual maximum and minimum temperature was 23°C and 3°C in July and 1°C and −18°C in January [34]. Trembling aspen, lodgepole pine, and Engelmann spruce are common tree species in several high elevation forests in this area and these species often form near-monotypic stands [35], [36], [37].

Four replicate blocks were identified where near-monotypic stands of aspen, pine, and spruce occurred in close proximity to one another (<200 m apart; Table 1). Stands of each tree species within a block shared similar elevation, aspect, slope, and bedrock, and the short distance among stands within a block ensured they had a similar macroclimate. However, there was variation in environmental conditions between the blocks, which were located 1–5 km from each other. The stands of each tree species varied in size, but in all cases stand area was at least hundreds of square meters and often much larger. At each sampling site the forest floor of each stand was always dominated by leaf litter derived from the dominant tree species in that stand (EA, HS personal observation), indicating that nearby stands of other tree species had little influence on the soil properties. The sampling sites were located within an area that was extensively burned in 1879 [38]. Forests returned after the fire, but the US Forest Service also planted lodgepole pine trees, which are not normally very abundant at this elevation in this region, to speed the development of a forested ecosystem [39].

**Table 1:** Location, elevation, and aspect of the blocks.

Block	Latitude (°)	Longitude (°)	Elevation (m)	Aspect
1	37.70991	107.75186	2980	SE
2	37.70621	107.74642	3000	W
3	37.71623	107.74388	3070	W
4	37.73158	107.69841	3270	SW

In October 2006 (i.e. the end of the growing season), *in situ* soil respiration rate, soil temperature (5 cm), and volumetric soil water content (0–10 cm) were measured at three sites (approximately 2 m apart in an equilateral triangle) within each stand using an infra-red gas analyzer and temperature probe (PP Systems, Amesbury, MA), and HydroSense soil moisture probe (Campbell Scientific, Logan, UT). Naturally senesced leaf litter was collected in litter traps between late August and early October 2006 from locations surrounding the soil measurement sites and litter C and N concentrations were determined on an elemental analyzer (LECO, St Joseph, MI). The forest products techniques for analysis of litter C fractions were used to determine litter lignin and cellulose content [40]. In addition, sets of three soil cores (3.4 cm diameter, 10 cm deep, ∼3 cm apart) were collected at the same time and location as the respiration measurements. One soil core from each set was placed in a Tullgren funnel for 72 hours for mesofaunal extraction into 70% ethanol [41], from which mites (identified to order) and collembolans were enumerated. One core was used for microfaunal extraction using the sugar centrifugation technique [42], from which nematodes (identified to family and feeding group) [43], tardigrades, and rotifers were enumerated; some nematode individuals (2.7%) could not be assigned to family due to physical damage. Nematode, tardigrade, and rotifer abundances were expressed on a soil dry weight basis. The third soil core was sieved (2 mm mesh) and used for all remaining soil measures, which included coarse (>1 mm diameter) and fine (<1 mm diameter) root biomass, soil pH, soil C and N content, inorganic N concentration, microbial biomass C and N, and microbial community composition. Roots were washed, sorted, dried (65°C for 48 h), and weighed. Soil pH was determined on 1∶2 soil∶deionized water slurry [44]. Total soil C and N content was determined on an elemental analyzer (LECO, St Joseph, MI). Soil ammonium and nitrate concentrations were determined via 1 M potassium chloride extraction and analyzed on an autoanalyzer (OI Analytical, College Station, TX). Microbial biomass C and N was determined via the chloroform fumigation-extraction procedure [45], [46] using a total organic carbon analyzer (Shimadzu, Columbia, MD). A 0.2 g subsample of sieved, homogenized soil was extracted using the MoBio PowerSoil DNA extraction kit (Solano, CA) following the manufacturer's instructions. The DNA was quantified using the Invitrogen Quant-It DNA kit and a Tecan Infinite M500 plate reader and diluted to 1 ng µl^−1^ for further analyses.

The relative abundance of bacterial 16S rRNA genes and the ITS region of fungal ssu rRNA genes were measured using quantitative PCR. The 16S gene was amplified with Eub 338 and Eub 518 primers, and the ITS gene was amplified with ITS 1 and 5.8S primers using conditions described by Fierer et al. [47] and three analytical replicates were measured for each sample. Ratios of rRNA are only indices of the relative abundance, and do not necessarily indicate biomass ratios.

To analyze fungal and bacterial community structure, we conducted a terminal restriction fragment length polymorphism (TRFLP) analysis using the primers sets ITS1f-FAM/ITS4r and 63f/1087r-FAM respectively. The T-RFLP technique provides an overall assessment of microbial community composition and diversity, but is not equivalent to any specific level of taxonomic resolution [48]. Since a single TRF can represent multiple species, changes in the relative of abundance of any TRF may not necessarily reflect changes in the relative abundance of particular taxonomic groups. However, changes in the composition of T-RFLPs represent changes in community composition. PCR reactions were run with 25 µl JumpStart REDTaq Ready Mix (Sigma-Aldrich, St Louis, MO, USA), 25 µM of each primer, 10 ng template DNA and 10 µl _dd_H_2_O. PCR reactions were run in a MyCycler Thermo Cycler (Bio-Rad, Hercules, CA, USA) with an annealing temperature of 55°C and 35 cycles. PCR products were cleaned using the Quick Clean 5M PCR Purification Kit (GenScript, Piscataway, NJ, USA). A 15 µl DNA aliquot was digested with *Msp*I (New England Biolabs, Ipswich, MA, USA) at 37°C for three hours. Digested PCR products were cleaned with the Quick Clean 5M PCR Purification Kit. Purified DNA (0.5 µl) was suspended with 5 µl Formamide and 0.5 µl 500 LIZ Size Standard (Applied Biosystems, Foster City, CA, USA). Electrophoresis was carried out in the GeneScan mode of an automated DNA sequencer (model 373, PE Applied biosystems) for up to 6 h using the following settings: 2500 V, 40 mA and 27 W. After electrophoresis, the size of each T-RF was determined in comparison with the Liz 500 internal length standard using the genescan analysis software provided by the manufacturer (PE Applied Biosystems). Profiles were analyzed using genescan genotyper program (PE Biosystems). T-RFLP peaks were aligned using the RiboSort software package [49], and peak heights were normalized to total peak height. Peak lengths smaller than 100 bp or larger than 600 bp were excluded. A consensus profile was created for each sample by including only peaks that were present in all three analytical replicates.

The effect of tree species identity and block, as well as their interaction, on the measured variables was tested using ANOVA. All faunal abundances, root biomass, soil C and N, ammonium concentration, and microbial biomass C and N were log transformed to meet assumptions of normality and homogeneity of variance. Where significant effects of tree species were observed, a post-hoc Tukey HSD test was used to determine which means were significantly different from one-another (*P* = 0.05). The relative abundance of different microbial TRFs and nematode families were analyzed by nonmetric multidimensional scaling (NMS) analysis, which produced an ordination score for the bacterial, fungal, or nematode community in each sample. The effect of tree species and block on microbial and nematode community structure was tested using ANOVA of the ordination scores of the three NMS axes that explained the most variance. Axis 1, 2, and 3 explained 29%, 21%, and 33% of fungal community variation, respectively, 18%, 35%, and 25% of bacterial community variation, respectively, and 16%, 11%, and 9% of nematode community variation, respectively.

## Results

### Plant properties

N concentrations in aspen litter were almost twice as high as in spruce or pine, whereas, pine litter had higher concentrations of C, cellulose, and lignin than either aspen or spruce (Table 2). Driven largely by differences in N concentration, aspen litter exhibited markedly lower C∶N than either coniferous species, while litter C∶N did not differ significantly between pine and spruce (Table 2). Aspen litter also had the lowest ratio of lignin∶N, while this measure was highest in pine litter (Table 2). Neither coarse (>1 mm diameter) or fine (<1 mm diameter) root biomass in the top 10 cm soil differed among stands of the three tree species (Table 2).

**Table 2:** Leaf litter quality and root biomass of tree species (mean±SE) and *F* and *P* values from ANOVA.

	Litter N (%)	Litter C (%)	Litter C∶N	Litter cellulose (%)	Litter lignin (%)	Litter lignin∶N	Fine root biomass (g m^−2^)*	Coarse root biomass (g m^−2^)*
Aspen	0.84±0.08^a^ †	49.4±0.4^a^	60.4±5.2^a^	42.1±3.0^a^	20.2±2.2^a^	24.1±1.2^a^	321±47	422±134
Pine	0.47±0.03^b^	52.0±0.3^b^	112.1±8.3^b^	59.0±1.0^b^	33.8±0.5^b^	73.2±5.7^b^	215±25	232±87
Spruce	0.41±0.01^b^	48.7±0.1^a^	118.3±4.2^b^	41.1±1.1^a^	19.2±0.7^a^	46.6±1.7^c^	249±28	319±62
Species	*F_2, 11_* = 19.0	*F_2, 11_* = 24.1	*F_2, 11_* = 23.9	*F_2, 11_* = 20.7	*F_2, 11_* = 33.9	*F_2, 11_* = 36.9	*F_2, 35_* = 1.8	*F_2, 35_* = 2.1
	*P* = 0.003	*P*<0.001	*P* = 0.001	*P* = 0.002	*P*<0.001	*P*<0.001	*P* = 0.204	*P* = 0.169
Block	*F_3, 11_* = 0.7	*F_3, 11_* = 0.9	*F_3, 11_* = 0.7	*F_3, 11_* = 0.3	*F_3, 11_* = 0.8	*F_3, 11_* = 0.3	*F_3, 35_* = 0.2	*F_3, 35_* = 0.8
	*P* = 0.585	*P* = 0.381	*P* = 0.587	*P* = 0.845	*P* = 0.532	*P* = 0.847	*P* = 0.900	*P* = 0.539
S×B	NA§	NA	NA	NA	NA	NA	*F_6, 35_* = 1.8	*F_6, 35_* = 1.8
							*P* = 0.172	*P* = 0.184

* Fine roots were<1 mm diameter; coarse roots were >1 mm diameter.

§ Not applicable since there was only one litter sample per stand.

† Different letters denote significant differences among tree species (*P* = 0.05).

### Soil properties

**Figure 1:**
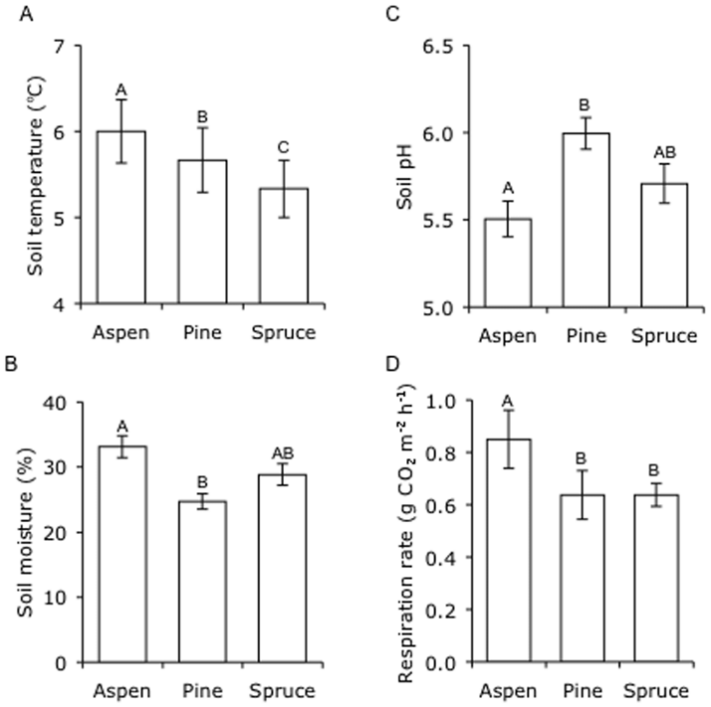
Soil physical and chemical properties and respiration rates in stands of three tree species. Soil (A) temperature, (B) moisture, (C) pH, and (D) respiration rates in stands of each species. Values are means±SE and letters denote significant differences among tree species (*P* = 0.05).

**Figure 2:**
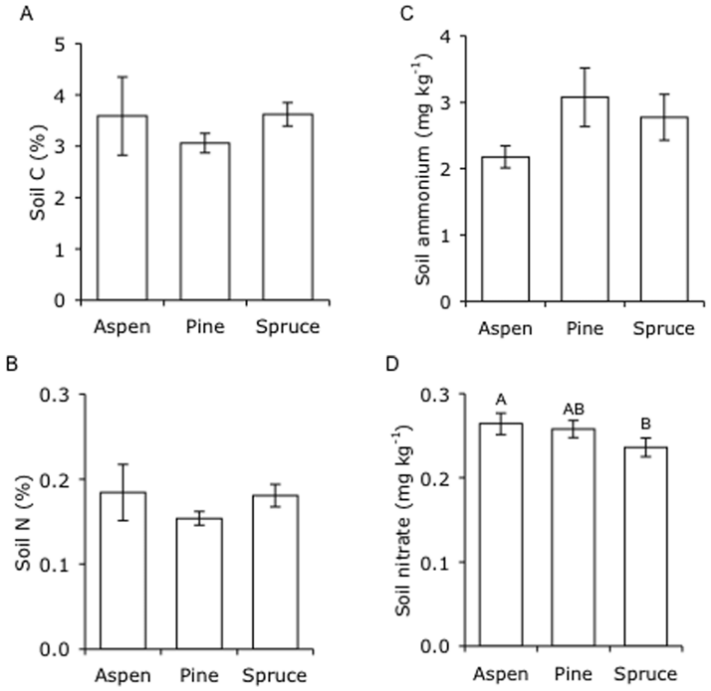
Soil carbon and nitrogen concentrations in stands of three tree species. Total soil (A) C, (B) N and (C and D) mineral N in stands of each species. Values are means±SE and letters denote significant differences among tree species (*P* = 0.05).

Soil temperature was highest in aspen (6°C) and lowest in spruce stands (5.3°C), while soil moisture was highest in aspen (33%) and lowest in pine stands (25%; Fig. 1, Table 3). All soils were acidic, ranging from 4.9 to 6.5, with the most acidic soils found in aspen stands and the least acidic soil in pine stands (Fig. 1, Table 3). Consistent with higher litter quality, soil temperature, and soil moisture in aspen stands, *in situ* soil respirations rates were 33% greater in aspen stands than in stands of pine or spruce (Fig. 1, Table 3). Total soil C, N, and ammonium (the dominant form of mineral N) concentrations did not differ among stands of the three tree species (Fig. 2, Table 3). However, nitrate concentrations were highest in aspen stands and lowest in spruce stands (Fig. 2d, Table 3).

**Table 3:** ANOVA output of the effect of tree species and block on soil properties and animal abundances.

	Species		Block		S×B	
	*F_2, 35_*	*P*	*F_3, 35_*	*P*	*F_6, 35_*	*P*
Temperature	22.2	<0.001	252.3	<0.001	10.5	<0.001
Moisture	6.8	0.011	1.9	0.177	1.3	0.300
pH	5.3	0.022	0.9	0.468	1.4	0.297
Respiration	5.5	0.020	10.2	0.001	4.6	0.012
C content	1.4	0.266	10.8	0.001	5.1	0.008
N content	1.3	0.307	9.3	0.002	2.9	0.053
Ammonium concentration	2.8	0.101	3.9	0.038	1.4	0.296
Nitrate concentration	6.2	0.014	12.6	<0.001	6.4	0.003
Microbial biomass C	1.3	0.325	4.9	0.027	3.6	0.043
Microbial biomass N	0.2	0.810	2.2	0.163	1.2	0.401
Fungi∶bacteria	1.9	0.206	3.2	0.077	1.4	0.300
Bacterial community (axis 1)*	1.2	0.344	7.0	0.012	1.8	0.224
Bacterial community (axis 2)*	9.4	0.008	7.5	0.011	2.0	0.184
Bacterial community (axis 3)*	24.1	<0.001	4.3	0.045	5.3	0.017
Fungal community (axis 1)*	2.8	0.115	5.1	0.029	3.4	0.056
Fungal community (axis 2)*	0.6	0.556	0.1	0.939	2.0	0.184
Fungal community (axis 3)*	5.5	0.032	2.2	0.167	0.8	0.570
Rotifer abundance	5.6	0.021	2.1	0.162	1.3	0.351
Tardigrade abundance	2.9	0.096	0.9	0.486	0.4	0.846
Nematode abundance	0.9	0.441	4.0	0.038	6.7	0.004
Nematode community (axis 1)*	1.2	0.323	5.8	0.011	6.5	0.003
Nematode community (axis 2)*	0.3	0.728	2.0	0.162	2.9	0.055
Nematode community (axis 3)*	15.3	<0.001	3.5	0.049	2.2	0.117
Collembola abundance	4.8	0.030	2.3	0.126	1.0	0.449
Oribatid abundance	0.2	0.802	1.3	0.315	0.6	0.697
Mesostigmatid abundance	4.4	0.036	1.5	0.260	0.8	0.558
Prostigmatid abundance	0.5	0.626	1.4	0.278	0.8	0.565

* Based on ordination scores for the three axes that explained the most variance in the NMS analyses.

### Soil biota

**Figure 3:**
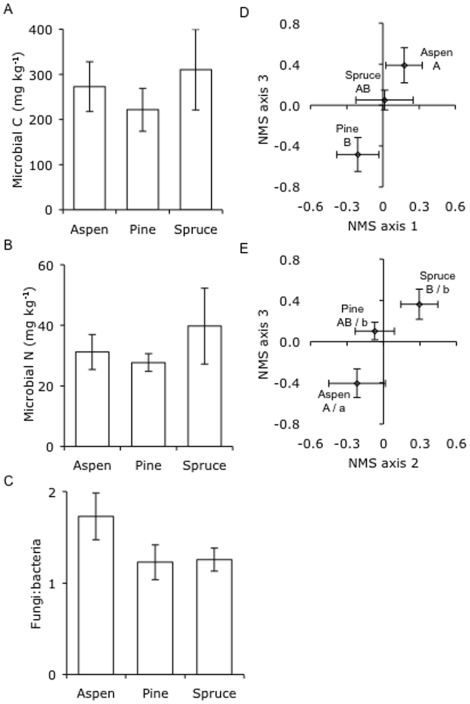
Microbial biomass and community composition in soil from stands of three tree species. Microbial biomass (A) C and (B) N, (C) fungal to bacterial rRNA gene ratio, and (D) fungal and (E) bacterial community composition (based on NMS ordination scores) in stands of each species. Values are means±SE and letters denote significant differences (*P* = 0.05) among tree species (upper and lower case letters refer to NMS axis 2 and 3, respectively, for bacterial community composition).

Microbial biomass C and N, and the ratio of fungal to bacterial rRNA genes, did not differ among the three tree species (Fig. 3, Table 3). However, both fungal and bacterial community composition differed among the tree species on at least one of the three NMS axes (Fig. 3, Table 3).

**Figure 4:**
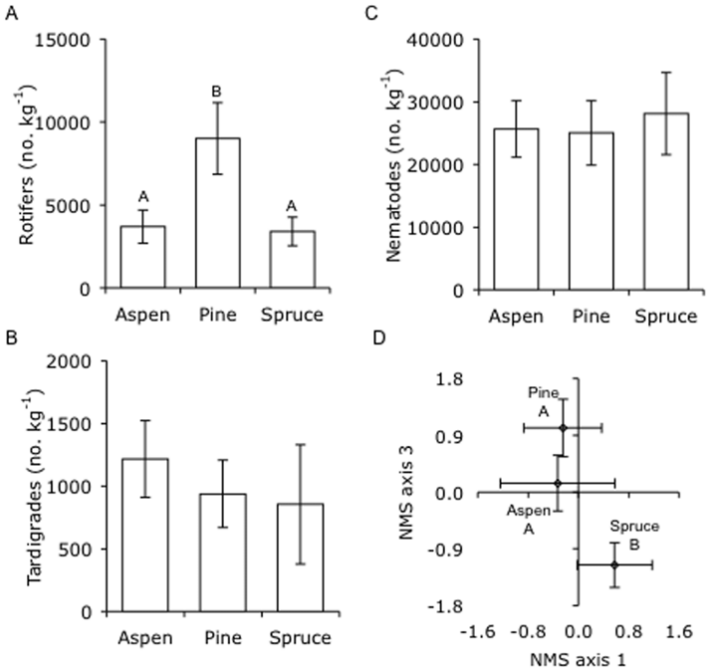
Microfaunal abundance and nematode community composition in soil from stands of three tree species. Total (A) rotifer, (B) tardigrade, and (C) nematode abundances, and (D) nematode community composition (based on NMS ordination scores) in soil from stands of three tree species. Values are means±SE and letters denote significant differences among tree species (*P* = 0.05).

Tree species also did not influence the total abundance of soil tardigrades or nematodes, or the abundance of any of the nematode feeding groups (i.e. plant parasites, root associates, bacterial feeders, fungal feeders, omnivores, or predators; Fig. 4, Table 3, Table 4). Similarly, the ratio of fungal- to bacterial-feeding nematodes did not differ significantly among the tree species (0.38±0.14, 0.74±0.34, and 0.41±0.06 in stands of aspen, pine, and spruce, respectively; *F_2, 35_* = 0.1, *P* = 0.948). However, rotifer abundance in the soil of pine stands was twice as high as in stands of aspen or spruce (Fig. 4, Table 3).

**Table 4:** Abundance of nematode feeding groups in stands of three tree species (mean±SE) and *F* and *P* values from ANOVA.

	Nematode abundance (no. kg^−1^)					
	Plant parasites	Root associates	Bacterial feeders	Fungal feeders	Omnivores	Predators
Aspen	5518±1919	7692±1660	6933±1430	2749±802	2130±461	382±123
Pine	6373±2606	5725±1324	8147±1854	2855±789	1509±365	342±165
Spruce	5252±2526	5906±1328	10277±2772	3669±1002	2835±605	171±108
Species	*F_2, 35_* = 0.3	*F_2, 35_* = 0.6	*F_2, 35_* = 1.8	*F_2, 35_* = 0.7	*F_2, 35_* = 2.3	*F_2, 35_* = 2.9
	*P* = 0.780	*P* = 0.559	*P* = 0.215	*P* = 0.513	*P* = 0.150	*P* = 0.096
Block	*F_3, 35_* = 5.3	*F_3, 35_* = 2.8	*F_3, 35_* = 0.8	*F_3, 35_* = 0.9	*F_3, 35_* = 1.1	*F_3, 35_* = 2.9
	*P* = 0.017	*P* = 0.090	*P* = 0.493	*P* = 0.477	*P* = 0.397	*P* = 0.084
S×B	*F_6, 35_* = 3.2	*F_6, 35_* = 1.9	*F_6, 35_* = 7.3	*F_6, 35_* = 2.3	*F_6, 35_* = 2.9	*F_6, 35_* = 3.7
	*P* = 0.044	*P* = 0.171	*P* = 0.003	*P* = 0.104	*P* = 0.059	*P* = 0.030

**Figure 5:**
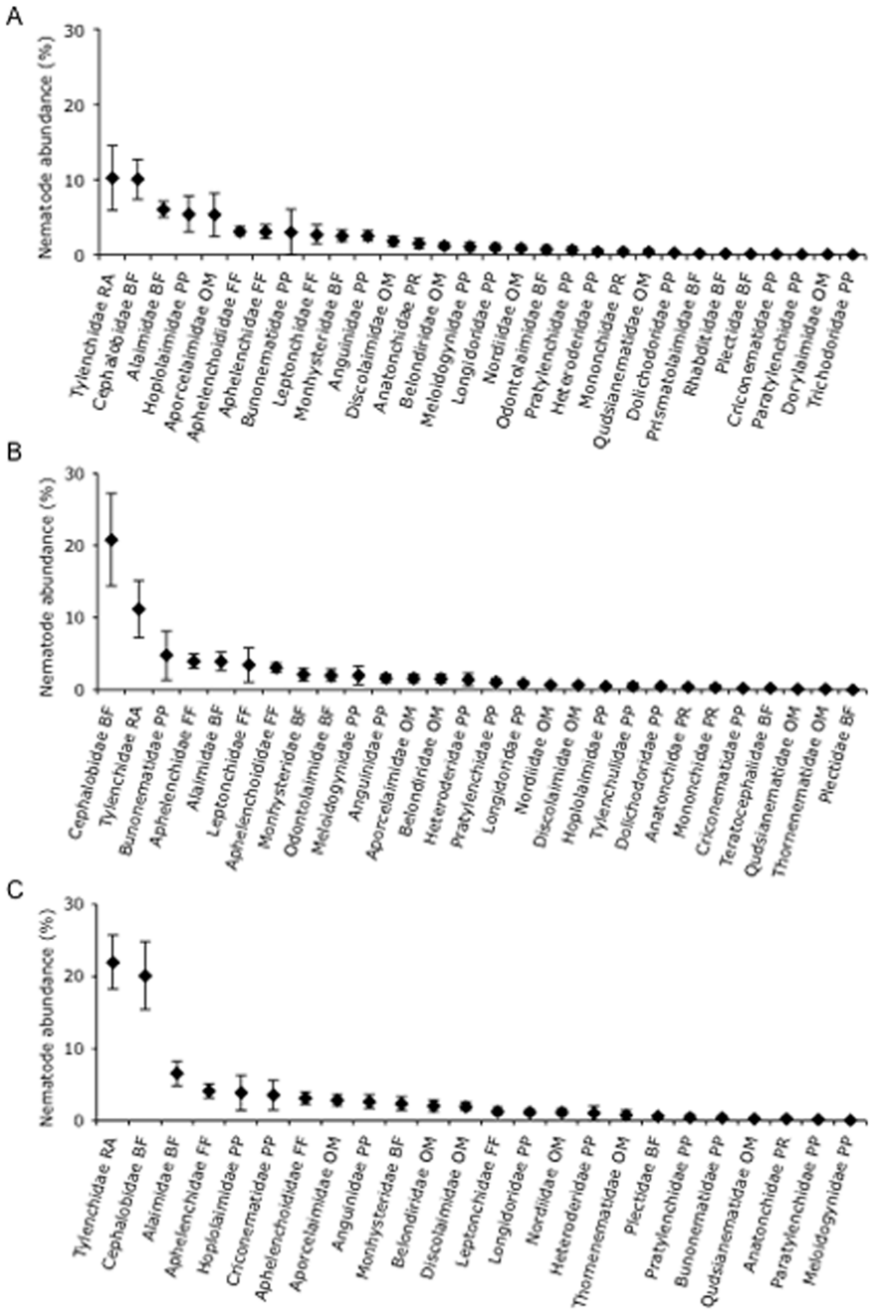
Relative abundance of nematode families in soil from stands of three tree species. Relative abundance of nematode families in order of mean abundance in stands of (A) aspen, (B) pine, and (C) spruce. Letters after each family denote feeding group: PP, plant parasite; RA, root associate, BF, bacterial feeder; FF, fungal feeder; OM, omnivore; and PR, predator. Values are means±SE.

Nematode community composition in spruce stands differed from the communities found in aspen or pine stands (Fig. 4d; Table 3). In total, 33 nematode families were identified across the three tree species. Cephalobidae (bacterial feeder) and Tylenchidae (root associate) were the dominant nematode families in stands of each species, together accounting for around 40%, 30%, and 20% of the nematode community in spruce, pine, and aspen stands, respectively (Fig. 5). Overall, 30 nematode families were found in aspen stands, 28 in pine stands, and 24 in spruce stands. Between one third and one half of the nematode families found beneath each tree species were rare (accounting for <1% of total nematode abundance). Twenty-two nematodes families occurred in stands of all three tree species, which included the eight most abundant nematode families beneath aspen, pine, and spruce (Fig. 5). Of the ten nematode families that were sufficiently abundant to analyze statistically, significant differences in abundance among tree species were only observed for the plant parasite Hoplolaimidae (*F_2, 35_* = 4.7, *P* = 0.034). The abundance of Alaimidae, Anguinidae, Aphelenchidae, Aphelenchoididae, Cephalobidae, Discolaimidae, Longidoridae, Monhysteridae, and Tylenchidae did not significantly differ among the three tree species.

**Figure 6:**
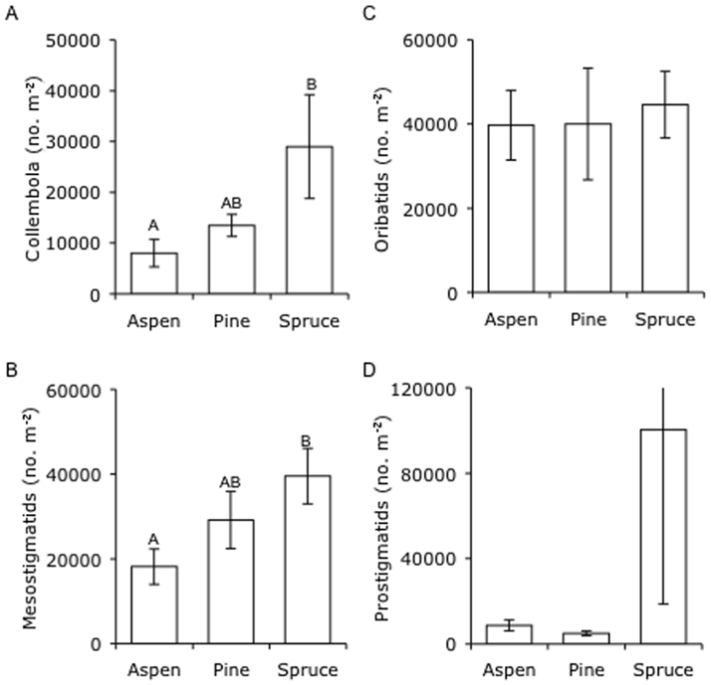
Mesofaunal abundance in soil from stands of three tree species. Abundance of (A) collembola, and (B) mesostigmatid, (C) oribatid, and (D) prostigmatid mites in stands of aspen, pine, and spruce. Values are means±SE and letters denote significant differences among tree species (*P* = 0.05).

The abundance of collembolans and mesostigmatid mites were lowest in aspen stands and highest in spruce stands (Fig. 6, Table 3). In contrast, the abundance of prostigmatid and oribatid mites did not differ among tree species stands (Fig. 6, Table 3). Mean prostigmatid mite abundance was considerably greater in spruce stands than in stands of the other species, although this was primarily driven by two samples from spruce stands that had particularly high abundances (990,000 and 139,000 m^−2^).

## Discussion

This study aimed to determine the effects of tree species on belowground properties and processes in high elevation forests by comparing stands of aspen, pine and spruce that occurred in close proximity to one another. We interpreted differences in belowground properties among stands of the three tree species to be the result of differences in tree species traits. It seems likely that the tree species were primarily driving the differences that we detected in the soil, rather than vise versa, since the lodgepole pine stands were planted [39] and we ensured that the macro-environmental conditions (slope, aspect, elevation, and bedrock) were similar among stands of the different tree species. It should be noted that significant block effects and tree species×block interactions were frequently observed indicating differences among the sites; however, we have not attempted to interpret these in the discussion since they were not the focus of this study. In addition, while a few of these results can easily be interpreted, such as higher soil temperatures at the lowest elevation site (7.2±0.2°C) and lower soil temperatures at the highest elevation site (4.3±0.2°C), most cannot easily be explained given the information that we have about these sites. For example, there is no obvious explanation why the abundance of plant parasitic nematodes was over 40% lower at block 1 than block 2 (blocks 3 and 4 were intermediate), therefore, we do not speculate about this.

Aspen stands had both warmer and wetter soils than coniferous stands. Warmer soils may have resulted from the relatively open canopy of aspen stands, which allows more sunlight to reach the soil. In addition, the aspen trees had already shed their leaves at the time of sampling, which further increased canopy openness and soil warming. Likewise, Hobbie et al. [9] observed species-specific effects of trees on mean annual soil temperature in a common-garden study that showed that broadleaf species typically had the warmest soil, while Norway spruce had the coldest soil, and pine species were intermediate. It seems likely that aspen stands had lower transpiration rates since they had senesced their leaves, whereas pine and spruce presumably had higher transpiration rates since both are evergreen, which may have contributed to increased soil moisture in aspen stands. In northwestern Ontario trembling aspen and jack pine were typically associated with drier soils than black spruce [50], supporting our notion that wetter soils under aspen stands than pine or spruce may have been due to our time of sampling (i.e. after leaf senescence).

The quality of aspen litter, measured as N concentration, C∶N or lignin∶N, was much higher than either coniferous species. Similar litter quality estimates for these tree species have been observed in other areas of the southern Rocky Mountains [26]. Decomposition and respiration rates are often positively related to litter quality, soil moisture (prior to saturation of the soil), and soil temperature [51], [52], [53], [54]. Therefore, the higher litter quality of aspen relative to pine and spruce, as well as higher soil temperature and moisture, likely contributed to faster respiration rates in soils from aspen stands.

Plant roots and soil microbes are directly responsible for the majority of soil respiration; however, neither differed in biomass among stands of the three species, suggesting that the differences in respiration rates result from differences in activity and/or differences in microbial community composition. Indeed, laboratory incubation studies in other ecosystems show that soil communities associated with different plant species differ in their respiration rate when incubated with leaf litter [55], [56], suggesting that the differences in fungal and bacterial community composition that we observed may have been partially responsible for differences in respiration rates.

Nematode communities are typically diverse and the large number of rare nematode taxa is a common feature of soils [57]. In our study, overall nematode community composition in spruce stands differed from the communities found in aspen and pine stands. However, only one family (Hoplolaimidae) out of the ten nematode families that could be analyzed statistically exhibited a difference in abundance among the tree species, being lowest in pine stands. Hoplolaimidae is a plant parasite [43], so given the direct trophic link with plants it is not surprising that it differed in abundance among the different tree species. However, two other plant parasites, Anguinidae and Longidoridae, and the root associate Tylenchidae did not differ in abundance among the tree species. The remaining nematode families that were statistically analyzed were bacterial feeders, fungal feeders, or omnivores [43] and as such did not have a direct trophic link to the tree species.

There was little evidence of a difference in the relative importance of the bacterial versus fungal energy channel among stands of the three tree species, despite differences in litter quality being associated with changes in the relative importance of these energy channels [58], [59]. Fungal∶bacterial rRNA gene ratios and the abundance of fungal- and bacterial-feeding nematodes did not significantly differ among the three tree species. However, collembolans, which are primarily fungal-feeders, were most abundant in stands of spruce and least abundant in stands of aspen, potentially indicating a shift towards the fungal energy channel in spruce stands. The relative importance of the fungal energy channel typically increases with decreasing quality of plant litter inputs [59], which is consistent with the differences in litter quality between aspen and spruce. Alternatively, the thicker litter layer of spruce stands (personal observation) may support a larger collembolan population due to increased habitat space [60].

Although differences in the soil biotic community among the tree species were observed, it is perhaps surprising that these differences were not more pronounced. Due to the large range of taxonomic groups we identified we could not classify any of them beyond family level, which likely masked much of the diversity of the community. Had it been possible to identify the soil organisms to species, which would have been a substantial undertaking across all these faunal groups, we suspect that greater differences in community structure would have emerged. In addition, it should be noted that the community structure of soil biota varies seasonally [61], [62], [63]; therefore, we may have observed larger differences in community composition among the tree species if we had sampled at a different date.

High elevation forests in the western USA face numerous environmental changes, including factors that operate at global, regional, and local scales [28], [29], [30], [31], [32], [33], [64]. Moreover, trembling aspen, lodgepole pine, and Engelmann spruce may exhibit species-specific responses to these environmental changes due to their differing natural histories, resulting in shifts in their relative abundance. For example, spruce and pine are susceptible to spruce bark beetle attack; therefore, changes in the frequency of beetle outbreaks, which may occur as a result of climate change [30], would likely alter the abundance of these species relative to aspen. Our study indicates that shifts in tree species occurrence will not only alter the appearance of these high elevation forests, but will also influence soil physical, chemical and biological properties in this ecosystem.
